# Circulating fibrocyte mobilization in negative pressure wound therapy

**DOI:** 10.1111/jcmm.13080

**Published:** 2017-02-17

**Authors:** Dezhi Chen, Yong Zhao, Zonghuan Li, Kangquan Shou, Xun Zheng, Pengcheng Li, Baiwen Qi, Aixi Yu

**Affiliations:** ^1^ Department of Orthopedics Zhongnan Hospital of Wuhan University Wuhan Hubei China

**Keywords:** diabetic wound, circulating fibrocyte, negative pressure wound therapy

## Abstract

Non‐healing diabetic wounds are difficult to treat. They also create heavy financial burdens for both patients and society. Negative pressure wound therapy (NPWT) has been adopted to treat intractable wounds and has proved to be effective. However, the mechanisms that underlie the effects of this treatment are not entirely understood. Circulating fibrocytes are unique haematopoietic‐derived stem cells that have been reported to play a pivotal role in wound healing. Here, we have investigated the effect of NPWT on fibrocyte mobilization and the role of fibrocyte mobilization in the healing of diabetic wounds during NPWT. We show that the NPWT group exhibited 2.6‐fold to 12.1‐fold greater numbers of tail vein‐injected PKH‐26‐labelled fibrocytes in the diabetic wound sites compared with the control group. We also demonstrate that the full‐thickness skin wounds treated with NPWT exhibit significantly reduced mRNA and protein expression, blood vessel density and proliferating cells when exogenous fibrocyte mobilization is inhibited. We speculate that systemic mobilization of fibrocytes during NPWT may be a mechanism for healing intractable wounds in a diabetic rat model experiment and that enhancement of cell mobilization may represent a potential treatment idea for intractable wound healing across all fields of surgery.

## Introduction

Non‐healing complex wounds are difficult to treat in patients with diabetes. They are associated with high economic burden as well as increased length and frequency of hospitalization. Adequate surgical debridement, effective antibiotic therapy, correction of metabolic abnormalities and proper wound management are essential for healing the intractable wounds of patients with diabetes 1, 2. NPWT is an adjuvant therapy that uses negative pressure to evacuate infected fluid from the open wound through a sealed dressing and a tube that is connected to suction 3, 4. This technique has enormous promise in treating battlefield casualties for whom access to first‐rate medical care may be limited. This is particularly true for personnel exposed to chemical agents, such as sulphur mustard, which have mechanisms of action that impair normal wound healing 5. The effectiveness of NPWT was also supported in the treatment of diabetic wounds by numerous prospective multicentred randomized controlled trials 6, 7, 8, 9, 10. However, the underlying mechanisms are not fully understood.

Circulating fibrocytes are a unique leucocyte subpopulation circulating in peripheral blood. They primarily reside in the bone marrow and can mobilize to enter the circulation. Fibrocytes are adherent cells that display a spindle‐shaped morphology and features of haematopoietic cells, which are considered to be a class of bone marrow‐derived stem cells 11. Although fibrocytes compose only 0.1% to 0.5% of peripheral blood leucocytes, they constitute 10% of the cells that infiltrate subcutaneously implanted wound chambers in mice 11.

Fibrocytes migrate to the wound sites, where they develop and differentiate during the early‐phase of healing and regulate the process of healing by producing important cytokines, chemokines and growth factors, secreting essential extracellular matrix (ECM) production and promoting angiogenesis. Wound healing is associated with blood vessel formation and active cell proliferation. The platelet endothelial cell adhesion molecule (PECAM) was used as an endothelial cell surface marker, which might reflect new blood vessel density following skin damages. Ki67, an indicator of cell proliferation, is currently a positive nuclear proliferation marker that has become reliable.

A prior study reveals that the systemic endothelial progenitor cells (EPCs), considered as markers of healing and repair, are mobilized during NPWT in patients with diabetic foot wounds 12. Therefore, we hypothesize that the number of fibrocytes in diabetic wounds is also up‐regulated during NPWT and that mobilized fibrocytes might play an important role in the healing of intractable wounds.

## Materials and methods

### Rats

Sprague‐Dawley (SD) rats (males, 4–5 weeks old) were purchased from the Animal Laboratory of centers for Disease Control and Prevention of Hubei Province (Wuhan, China). All experiments were approved by the Institutional Animal Care and Use Committee of Wuhan University, and the animal procedures were performed in strict accordance with institutional and national guidelines 13. All efforts were made to minimize suffering.

### Diabetic rat model

Young male rats were used as study subjects. After overnight fasting, all rats were given a 60 mg/kg intraperitoneal injection of freshly dissolved streptozotocin (STZ, Sigma‐Aldrich, St. Louis, MO, USA) in 50 mmol/l citrate buffer (pH 4.0). After 72 hrs, glucose levels of tail vein blood were measured using a glucometer (Glucometer Elite XL; Bayer, Elkhart, IN, USA) and rats with serum glucose levels exceeding 16.7 mmol/l were selected for further observation. Diabetes mellitus was induced again in the rats that failed to reach the glycaemic standard by injection of 20 mg/kg STZ. Their serum glucose levels were then monitored weekly. A portion of these qualified adult rats was used for blood collection and cell isolation 7–9 weeks later. The remainder were used for *in vivo* study.

### Cell isolation and culture

Diabetic fibrocytes were isolated from peripheral blood and cultured as previously described 14, 15. Briefly, PBMCs were isolated from diabetic rat blood (heparinized) obtained by cardiac puncture following isoflurane inhalation. Blood sample was mixed with phosphate‐buffered saline (PBS) and layered over Ficoll/Paque (GE Healthcare, Piscataway, NJ, USA) and centrifuged according to the manufacturer's protocol. Cells were cultured in Dulbecco's modified Eagle's medium (DMEM) supplemented with 20% FBS (Tianhang Biological Technology Inc., Hangzhou, China), penicillin, streptomycin and L‐glutamine at 37°C in a humidified atmosphere with 5% CO_2_ in air. After 72 hrs’ incubation, non‐adherent cells (largely T cell) were removed by gentle aspiration, and media were replaced. For subculture, the adherent cells were detached with cold trypsin/EDTA (Gino biomedical technology Inc., Hangzhou, China) and passaged at a ratio of 1:2 plates when cells grew to 70–80% confluence and cultured in the same condition for up to 28 days. The medium was refreshed every 3 days. Fibrocytes were identified at day 28 as CD45 (+), αSMA (+) and Col‐I (+) by flow cytometry.

### Fibrocyte migration using a diabetic wound model

Thirty‐six diabetic rats were randomized into three groups: the NPWT group, NPWT/antagonist group and control group (*n* = 12/each group). The dorsa of rats were clipped and depilated 24 hrs before the experiment. Rats were anaesthetized with pentobarbitone sodium (40 mg/kg) 5 min. before surgery. Cultured fibrocytes at day 28 were labelled with a membrane‐inserting red dye (PKH‐26, Sigma‐Aldrich) following the manufacturer's protocol. PKH‐labelled cells (7.5 × 10^5^) in 100 μl PBS were injected into the tail veins of SD rats. Immediately, a full‐thickness skin wound (1.5 × 1.5 cm) was created in the mid‐dorsal area of each recipient rat. A medical foam (VSD Medical Technology Inc., Wuhan, China) covered the wound and was exposed to continuous 125 mmHg suction produced by a vacuum‐assisted device in the NPWT group (*n* = 12). In the control group (*n* = 12), moist gauze dressings covered the wounds and were changed daily.

In the NPWT/antagonist group (*n* = 12), PKH‐labelled fibrocytes were incubated in the AMD3100 (CXCR4‐specific antagonist; Selleck) solution of 25 ng/ml concentration in the CO_2_ incubator for 1 hr and then were centrifuged to obtain pre‐treated fibrocytes. These cells were mixed with PBS, and an equal amount of treated cells (7.5 × 10^5^) in 100 μl PBS were injected into the rat tail veins. Subsequently, wound models and NPWT were performed as described above.

### Sampling and detection

During 7 days of treatment, three rats in each group were killed at each specified time‐point (12 hrs, 2 days, 4 days and 7 days post‐surgery). The wound tissues were harvested and examined for the presence of fluorescent fibrocytes by microscopic analysis of thin frozen sections and quantitative flow cytometric analysis following proteolytic digestion. The wound tissues harvested at day 7 were also examined for mRNA expression by RT‐PCR assay and protein expression by Western blot assay. Angiogenesis and cell proliferation were analysed by immunohistochemistry analysis of paraffin‐embedded sections.

### Quantitative flow cytometric analysis

For quantitative flow analysis, excised wound tissues from each sample were chopped into small fragments and incubated for 30 min. at 37°C in 2 ml D‐hanks solution containing 1 mg/ml collagenase I. Equal volume of low sugar DMEM containing 10% FBS terminated the digestion, and the obtained cell suspension was filtered and centrifuged. The resulting single‐cell suspension was examined by flow cytometry to determine the ratio of fluorescent fibrocytes to total cells in wound sites.

### Immunohistochemistry analysis for Ki‐67 and PECAM‐1

Paraffin‐embedded sections were deparaffinized, rehydrated and boiled in a microwave oven in EDTA buffer (pH 8.0) for antigen retrieval. Primary antibodies against mouse PECAM‐1 (1:75; Abcam, Cambridge, UK) and rabbit Ki‐67 (1:300; Abcam) were incubated at 4°C overnight. Sections were washed and incubated with horseradish peroxidase‐conjugated goat antimouse (1:200; Aspen Inc., Wuhan, China) and goat anti‐rabbit (1:200; Aspen Inc.) IgG secondary antibodies for 50 min. After being washed with PBS, sections were incubated with freshly prepared 3′3‐diaminobenzidine (DAB) solution (Dako, Copenhagen, Denmark). Colour development was controlled under an optical microscope. Subsequently, sections were counterstained with haematoxylin stain to visualize nuclei. All images were captured using a light microscope (Olympus, Tokyo, Japan).

To quantify angiogenesis, five randomly selected fields were evaluated at 200× magnification and the neovascular area (PECAM‐1‐positive cells) was measured using Image J (NIH, Bethesda, MD) and was expressed as a percentage of the PECAM‐1‐positive area to the total image area.

To quantify cell proliferation, high‐power digital images of Ki‐67‐stained wound sections were obtained at 200× magnification. The number of Ki‐67 positive cells in the image area was counted and expressed as a ratio of proliferating nuclei (Ki‐67 positive) to total nuclei in the wound areas.

### RT‐PCR assay

RNA was extracted from frozen tissue samples (harvested at 12 hrs, 2 days, 4 days and 7 days) using TRIzol Reagent (Invitrogen™, Carlsbad, CA) following the manufacturer's protocol. After DNase digestion was performed, cDNA was synthesized using PrimeScript™RT reagent Kit with gDNA Eraser (Takara Bio, Shiga, Japan). The primer sequences were described in Table 1.

**Table 1:** Primer sequences for qRT‐PCR

Gene	Forward primer	Reverse primer
PDGF‐A	AAGACCAGGACGGTCATTTACG	CTAACCTCACCTGGACCTCTTTC
TGF‐β1	GTGGCTGAACCAAGGAGACG	AGGTGTTGAGCCCTTTCCAG
SDF‐1	GTAAGCCAGTCAGCCTGAGCTAC	GGATCCACTTTAATTTCGGGTC
α‐SMA	CACCATCGGGAATGAACGCT	CTGTCAGCAATGCCTGGGTAC
Col‐I	CCGTGACCTCAAGATGTGCC	GAACCTTCGCTTCCATACTCG
VEGF‐A	ATCTTCAAGCCGTCCTGTGTG	AGGTTTGATCCGCATGATCTG
MCP‐1	CAATGAGTCGGCTGGAGAACT	ACAGAAGTGCTTGAGGTGGTTG
MIP‐1α	GACTGCCTGCTGCTTCTCCTAT	AGTGATGTATTCTTGGACCCAGG
GAPDH	CGCTAACATCAAATGGGGTG	TTGCTGACAATCTTGAGGGAG

The real‐time RT‐PCR was performed using the StepOne™ Real‐Time PCR System (Life Technologies, Gaithersburg, MD). Thermal cycling was performed as follows: an initial degeneration at 95°C for 15 sec., and then annealing at 58°C for 20 sec., extension at 72°C for 45 sec. Each sample was run in triplicate.

### Western blot assay

Total proteins in each sample on day 7 extracted using the protein extracting reagent RIPA (Aspen Inc.) following the manufacturer's protocol. The protein concentration was determined using the BCA kit (Aspen Inc.). Equal amounts of proteins from each sample were separated by SDS‐PAGE, transferred to PVDF membranes (Millipore Corporation, Billerica, MA) and blocked with 5% milk for 1 hr at room temperature. The membranes were incubated overnight at 4°C with rabbit anti‐GAPDH antibody (1:10,000; Abcam Inc.), rabbit anti‐VEGF‐A antibody (1:1000; Bioss Biotechnology Inc., Beijing, China), rabbit anti‐MIP‐1α antibody (1:1000; Abcam Inc.), rabbit anti‐α‐SMA antibody (1:5000) (TDY Biotechnology Inc., Beijing, China) and mouse anti‐collagen I antibody (1:500; Abcam Inc.). Finally, the membranes were subsequently incubated with the horseradish peroxidase‐conjugated goat anti‐rabbit IgG antibody (KPL) and goat antimouse IgG antibody (KPL) as secondary antibody at 1:10,000 for 30 min. The protein bands were detected by ECL chemiluminescence and quantified by AlphaEaseFC (Alpha Innotech, San Leandro, CA) software.

### Statistical analysis

Data were presented as the mean ± S.D. Data comparisons were performed by *t*‐tests, two‐way anova and Bonferroni post‐tests (SPSS 18.0; SPSS Inc., Chicago, IL, USA). A *P* value < 0.05 was considered statistically significant.

## Results

### Cell culture

**Figure 1:**
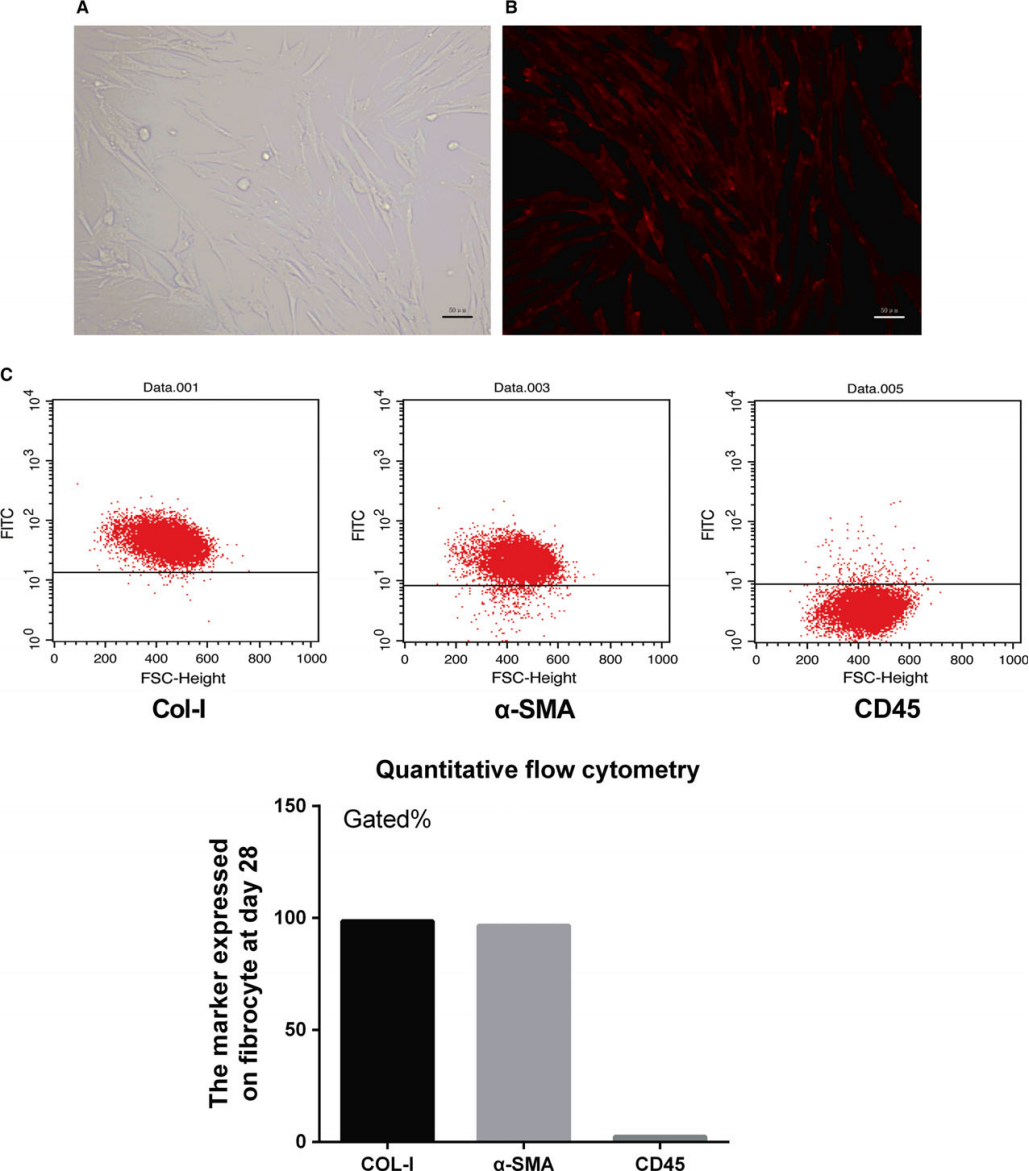
Cell culture. Fibrocytes were isolated from circulation and were cultured in DMEM at 37°C in a humidified atmosphere with 5% CO_2_ in air. After culturing for 28 days, these cells were thin with elongated spindle shapes (**A**, magnification: 100×); fibrocytes on day 28 were labelled with PKH‐26 following the manufacturer's protocol and then observed under fluorescence microscope (**B**, magnification: 100×). Flow cytometry showed that fibrocytes express markers of both haematopoietic and stromal cells. The level of CD45^+^ cells at day 28 was rather low; however, the proportion of Col‐I^+^ and αSMA ^+^ cells increased dramatically to more than 95 percent (**C**).

Under an optical microscope, fibrocytes isolated from rat peripheral blood proliferated and displayed a mesenchymal morphology showing an elongated spindle shape in the late stage (Fig 1A). Under fluorescence microscope, cultured fibrocytes at day 28 were labelled with a membrane‐inserting red dye PKH‐26, showing large amounts of red spindle cells with many overlaps (Fig 1B). Flow cytometry shows that fibrocytes express markers of both haematopoietic and stromal cells. Consistent with the previous study 16, the level of haematopoietic marker CD45^+^ on fibrocytes was decreased, whereas the expression of ECM components (Col‐I^+^ and αSMA^+)^ was up‐regulated in the late stage (Fig 1C).

### Quantitative flow cytometric analysis

**Figure 2:**
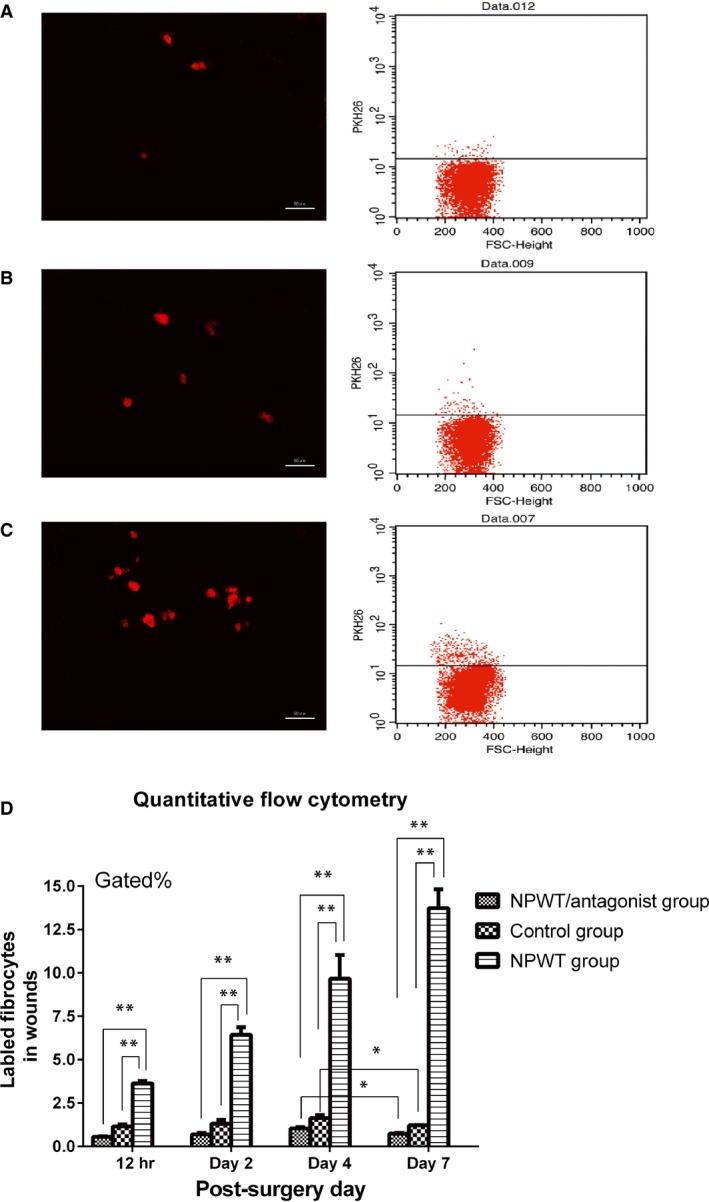
Quantitative flow cytometric analysis. At each specified time‐point (12 hrs, 2 days, 4 days and 7 days), the wound sites were excised and examined for the presence of fluorescent fibrocytes by microscopic analysis of thin frozen sections and quantitative flow cytometric analysis following proteolytic digestion. (**A**) Microscopic analysis and quantitative cytometric analysis of wound tissues at day 2 in the NPWT/antagonist group; (**B**) microscopic analysis and quantitative cytometric analysis at day 2 in the control group; (**C**) microscopic analysis and quantitative cytometric analysis at day 2 in the NPWT group. These showed that NPWT‐treated wounds exhibited significantly more exogenous fibrocytes during the early‐phase of wound healing compared with both the control and NPWT/antagonist groups; (**D**) statistical comparison of fibrocyte recruitment to the wounds at different time‐points among the three groups (*P < 0.05, **P < 0.01).

As shown in Figure 2, quantitative flow cytometry revealed that PKH‐labelled fibrocytes could be found in the wound sites as early as 12 hrs in all three groups. The NPWT group exhibited 2.6‐fold to 12.1‐fold increases of labelled cells compared to the control group and 5.7‐fold to 21.3‐fold as many cells as the NPWT/antagonist group at different time‐points (12 hrs, 2 days, 4 days and 7 days). Additionally, we found that the number of labelled fibrocytes recruited to the wounds increased before day 4 in all three groups. However, the number began to decrease from day 4 in the NPWT/antagonist group and control group, whereas it was still increasing in the NPWT group.

### Cell proliferation and angiogenesis in wound sites

**Figure 3:**
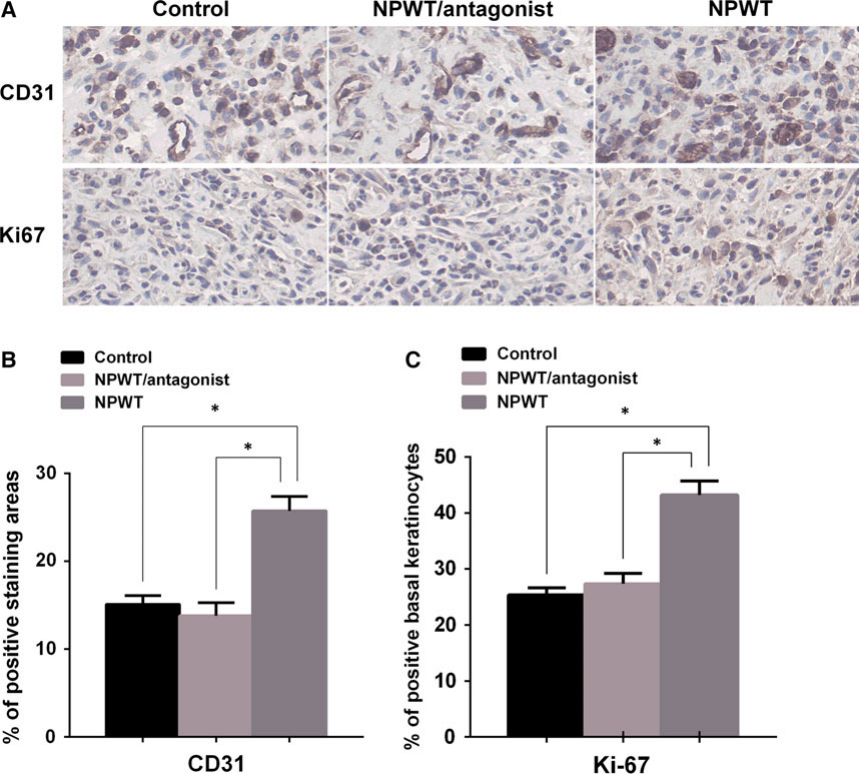
Cell proliferation and angiogenesis in wound sites. (**A**) Wound tissues at day 7 were stained for neovascularization (PECAM‐1) (upper panel, magnification: 200×) and proliferating cells (Ki‐67) (lower panel, magnification: 200×). (**B**) NPWT‐treated wounds exhibited significant increases in calculated blood vessel density when compared with both control and NPWT/antagonist groups on day 7 (*P < 0.05). (**C**) NPWT‐treated wounds exhibited significant increases in cell proliferation index when compared with both control and NPWT/antagonist groups on day 7 (*P < 0.05).

To investigate whether the change in the number of exogenous fibrocytes recruited to the damaged tissues affected the healing of diabetic wounds, immunohistochemistry of wounds on day 7 was performed and quantified. PECAM‐1 (CD31) was selected to stain endothelial cells (Fig 3A, upper panel), and proliferating cells were stained for Ki‐67 (Fig 3A, lower panel). Compared to the NPWT group, the control group showed a significant decrease in blood vessel density and cell proliferation. The NPWT/antagonist group showed the same differences compared to the NPWT group (Fig 3B and C).

### mRNA expression and protein expression

**Figure 4:**
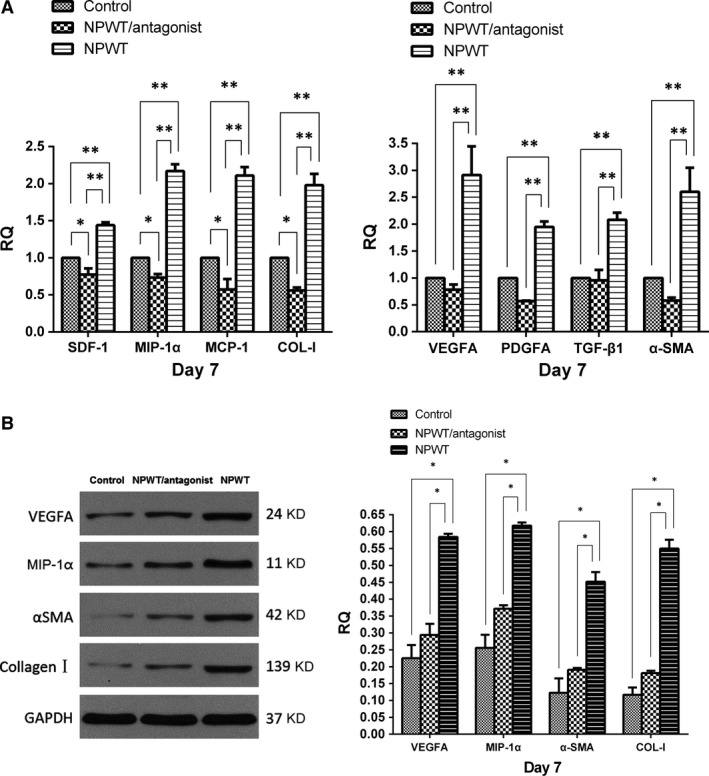
RT‐PCR and Western blot analysis. Excised wound tissues at day 7 in the three groups were used to examine the expressions of wound healing‐related factors. (**A**) RT‐PCR assay. Compared with the control and NPWT/antagonist groups, the NPWT group increased the expression levels of chemokines (SDF‐1, MIP‐1α and MCP‐1), growth factors (VEGF‐A, PDGF‐A and TGF‐β), α‐SMA and Col‐I. Additionally, wounds in the NPWT/antagonist group exhibited lower levels of SDF‐1, MIP‐1α, MCP‐1 and Col‐I when compared with the control group (*P < 0.05, **P < 0.01). (**B**) Western blot assay. The NPWT group exhibited higher levels of VEGF‐A, MIP‐1α, α‐SMA and Col‐I on day 7 compared with the control and NPWT/antagonist groups (*P < 0.05).

After tail vein injection of labelled fibrocytes, the NPWT group developed higher expression levels of chemokines (SDF‐1, MIP‐1α, MCP‐1), growth factors (TGF‐β1, VEGF‐A, PDGF‐A) and extracellular matrix proteins (Col‐I, α‐SMA) at day 7 compared with the control and NPWT/antagonist groups (Fig 4A). Western blot assay also showed that the level of VEGF‐A, MIP‐1α,α‐SMA and Col‐I on day 7 was significantly up‐regulated in NPWT group when compared with the other two groups (Fig 4B).

## Discussion

It is well known that NPWT is a well‐established therapy for treating traumatic wounds, general surgical wounds and diabetic wounds. However, the underlying mechanisms are less obvious. In this study, we provide new evidence that fibrocyte mobilization may play an important role in the process of NPWT‐treated diabetic wound healing. This is supported by the following: a) NPWT induced systemic mobilization of exogenous fibrocytes to the diabetic wounds and exhibited faster healing than treatment with moist gauze dressings and b) mobilization of exogenous fibrocytes was inhibited by AMD3100, thereby slowing the healing of diabetic wounds during NPWT in response.

In this study, we employed the signature surface marker profile‐CD45 ^+^/collagen I ^+^/αSMA^+^ to identify fibrocytes. The cell morphology changed to a spindle shape and started proliferating as the immature precursors adhered to the culture plate, a finding consistent with previous studies 14, 16. Fibrocytes derived from peripheral blood migrate towards damaged tissues in response to chemokine signals 14, 17, 18, 19, 20, 21. Chemokine receptors expressed on the surface of fibrocytes include CXCR4, CCR7, CCR3 and CCR5 14. Secondary lymphoid chemokine (SLC), a ligand of the CCR7 chemokine receptor, acts as a potent stimulus for fibrocyte chemotaxis *in vitro* and for the migration of injected fibrocytes to sites of cutaneous tissue injury *in vivo* 14. Previous studies have revealed that the SDF‐1/CXCR4 axis is not only involved in the invasion and metastasis of malignant tumours 22, 23 but also critical to the migration of stem cells 24, 25. We suggested that the SDF‐1/CXCR4 axis might also have a role in the migration of fibrocytes to wound sites.

AMD3100 can disrupt the binding of SDF‐1 to the CXCR4^+^ cells to inhibit cell chemotaxis *in vitro* 26, 27, 28. Accordingly, we suggested that AMD3100 might inhibit fibrocyte migration to the wound sites in a diabetic rat model experiment. However, application of this inhibitor met with some difficulties. Daily intravenous or intraperitoneal injection of AMD3100 would effectively mobilize haematopoietic stem and progenitor cells into peripheral blood due to reversible disruption of SDF‐1/CXCR4 binding. It would thus contribute to angiogenesis and accelerate wound healing 29, 30, 31. Additionally, daily injection of AMD3100 into the wound sites could not be performed because of the medical foam and membrane overlying the wound in this study. Therefore, we used exogenous, PKH‐labelled fibrocytes to indirectly observe the influence of NPWT on fibrocyte mobilization in this study. Note that the dosage of AMD3100 used in this study was small, and even less AMD3100 was used to prevent binding of CXCR4 to enter circulation, which might not be able to influence wound healing to affect the confirmation of our conclusion.

Flow cytometric analysis showed that PKH‐labelled fibrocytes were found as early as 12 hrs post‐injection and that the NPWT group exhibited significantly more labelled fibrocytes in the wound sites when compared with the control group at different time‐points. These findings suggest that NPWT induces the mobilization of circulating fibrocytes that migrate to the wound sites at the early stage of healing. Exogenous fibrocyte mobilization in the NPWT/antagonist group was markedly inhibited by AMD3100, indicating the importance of the SDF‐1/CXCR4 axis to the migration of injected fibrocytes to damaged tissues in diabetic rats. However, there was no significant difference in the level of labelled fibrocytes between the NPWT/antagonist group and the control group. This might be because fibrocytes are mobilized not only by increased chemokines around the wound tissue 32 but also by various other factors including increased blood flow, removal of oedema and mechanical stress in the bed of the wound produced by suction. Additionally, the number of labelled fibrocytes in the wounds declined after day 4 in both the NPWT/antagonist and control groups, whereas it continued to increase in the NPWT group, showing a longer period of fibrocyte accumulation in the diabetic wounds. However, the mechanisms underlying the effect are less obvious.

NPWT induced the mobilization of fibrocytes and promoted the healing of diabetic wounds. The effect of NPWT was markedly reduced when there was an inhibition in fibrocyte mobilization. This effect is consistent with a previous study showing that fibrocytes play a pivotal role in the healing of diabetic wounds 16 and that mobilized fibrocytes might contribute to the effectiveness of NPWT in a diabetic rat experiment. One limitation of our study was that only the early‐phases of NPWT were analysed. Therefore, we could not confirm the effect of prolonged NPWT on fibrocyte mobilization.

Changes in wound biochemistry and mechanical deformation alter the local environment through the process of mechanotransduction, that is the conversion of a mechanical stimulus into cellular biomechanical signals utilizing differential gene expression and protein secretion. *In vitro*, stretching increases human fibroblast growth and migration. In the animal wound closure model, NPWT increased the expression of ECM proteins, chemokines and growth factors. The fibrocyte mobilization that was induced during NPWT appears to have been caused by negative pressure strain.

Previous studies have demonstrated that chronic wound states in diabetes are correlated with the deficiency of functional stem cells, dysfunctional cellular response and insufficient cell proliferation in the injured tissues. In this setting, diabetic fibroblasts display selective impairments in discrete cellular processes critical for tissue repair including cellular migration, VEGF production and the response to hypoxia 33, 34, 35, 36, 37, 38, 39. In the diabetic rat experimental model, NPWT was effective in improving wound healing. This was likely caused by more extraneous and endogenous fibrocytes being mobilized and recruited to compensate for the dysfunctional resident dermal fibroblasts and provided an additional, renewable source of functional progenitor cells to promote wound healing.

The fibrocytes that were mobilized and recruited to the wound sites during NPWT contributed directly to the population of mature fibroblast‐ and myofibroblast‐like cells to produce more collagen and α‐SMA, which could enhance wound contraction. Furthermore, fibrocytes were reported to secrete several proangiogenic factors including VEGF, bFGF, IL‐8, PDGF and haematopoietic growth factors that promote endothelial cell migration, proliferation and tube formation 40. The proposed effects of fibrocytes might explain the mechanism of neovascularization during NPWT. A previous study revealed that microscopic keratinocytes in both active proliferation and macroscopic re‐epithelialization were stimulated in fibrocyte‐treated wounds, which was consistent with significant increases in levels of FGF‐7 16. We speculate that increased levels of FGF‐7 might promote keratinocyte proliferation and re‐epithelialization in the wound sites. However, the expression of FGF‐7 was not measured in this study.

Increased fibrocytes in the wounds were also associated with enhanced gene expression of many proinflammatory chemokines, suggesting that they contribute to the inflammatory process and amplify an inflammatory response. Moreover, the markedly up‐regulated expression of the potent mononuclear cell chemokine (MCP‐1 and MIP‐1α) might result in uptake of more bone marrow‐derived, blood‐borne cells critical to healing of the wound areas. TGF‐β1 and PDGF‐A are the most potent stimulators of wound healing 41 and might act in concert to regulate fibroblast migration, proliferation, angiogenesis and ECM production 42, 43. Taken together, injected fibrocytes contribute to diabetic wound healing not only by direct participation (fibrocytes migrate to wounds where they then develop and differentiate) but also by indirect regulation. This indirect regulation affects the proliferation and inflammatory responses and the functions of dermal fibroblasts and cells through the elaboration of chemokines, cytokines and growth factors.

In summary, this study demonstrates that fibrocyte mobilization is induced by NPWT to accelerate wound healing by stimulating wound re‐epithelialization, contraction and angiogenesis in a diabetic rat model. Fibrocytes were reported to play a pivotal role in wound healing and tissue repair processes. However, fibrocyte transplantation was limited in clinical practice due to the rarity of this cell population in circulation and non‐proliferating features 44. Furthermore, wounds treated with a combination of NPWT and cell therapy exhibited markedly accelerated healing when compared with those treated only with cell therapy. Therefore, it may be important for future research to pay more attention to the process of fibrocyte mobilization and recruitment to the wound sites and to utilize the combined treatment of NPWT and cell therapy to accelerate healing of non‐healing complex wounds. It should be noted that this investigation is preliminary. Future work is needed to evaluate fibrocyte mobilization in NPWT over longer time periods.

## Author contributions

AXY and BWQ conceived and designed the study. DZC, YZ, ZHL, KQS and XZ performed the experiments. DZC, PCL and XZ performed the statistical analysis. DZC, YZ and ZHL wrote the manuscript. AXY, BWQ, DZC, XZ, PCL and KQS reviewed and edited the manuscript. All authors read and approved the manuscript.
